# PPARGC1A Gene Promoter Methylation as a Biomarker of Insulin Secretion and Sensitivity in Response to Glucose Challenges

**DOI:** 10.3390/nu12092790

**Published:** 2020-09-11

**Authors:** José L. Santos, Bernardo J. Krause, Luis Rodrigo Cataldo, Javier Vega, Francisca Salas-Pérez, Paula Mennickent, Raúl Gallegos, Fermín I. Milagro, Pedro Prieto-Hontoria, J. Ignacio Riezu-Boj, Carolina Bravo, Albert Salas-Huetos, Ana Arpón, José E. Galgani, J. Alfredo Martínez

**Affiliations:** 1 Department of Nutrition, Diabetes and Metabolism, School of Medicine, Pontificia Universidad Católica de Chile, Santiago 8331150, Chile; rodrigo.cataldo_buscunan@med.lu.se (L.R.C.); jnvega@uc.cl (J.V.); p.mennickent@gmail.com (P.M.); ragallegosc@gmail.com (R.G.); carobravo.vasquez@gmail.com (C.B.); jgalgani@uc.cl (J.E.G.); 2 Instituto de Ciencias de la Salud, Universidad de O’Higgins, Avenida Libertador Bernardo O’Higgins 611, Rancagua 2841935, Chile; bjkrause@gmail.com (B.J.K.); francisca.salas@uoh.cl (F.S.-P.); 3 Department of Nutrition, Food Sciences and Physiology, Centre for Nutrition Research, University of Navarra, 31008 Pamplona, Spain; fmilagro@unav.es (F.I.M.); jiriezu@unav.es (J.I.R.-B.); aarpon.1@alumni.unav.es (A.A.); jalfmtz@unav.es (J.A.M.); 4 Centro de Investigación Biomédica en Red de la Fisiopatología de la Obesidad y Nutrición (CIBERobn), Instituto de Salud Carlos III, 28029 Madrid, Spain; 5 IdiSNA, Navarra’s Health Research Institute, 31008 Pamplona, Spain; 6 Facultad de Ciencias de la Salud, Universidad SEK, Santiago 8340363, Chile; pedro.prieto@usek.cl; 7 Andrology and IVF Laboratory, Division of Urology, Department of Surgery, University of Utah School of Medicine, Salt Lake City, UT 84108, USA; albert.salas@utah.edu; 8 Departamento de Ciencias de la Salud, Nutrición y Dietética, Facultad de Medicina, Pontificia Universidad Católica de Chile, Santiago 781000, Chile; 9 IMDEA-Food, 28049 Madrid, Spain

**Keywords:** methylation, insulin sensitivity, insulin secretion, biomarker, gene variant, PPARGC1A gene

## Abstract

Methylation in CpG sites of the PPARGC1A gene (encoding PGC1-α) has been associated with adiposity, insulin secretion/sensitivity indexes and type 2 diabetes. We assessed the association between the methylation profile of the PPARGC1A gene promoter gene in leukocytes with insulin secretion/sensitivity indexes in normoglycemic women. A standard oral glucose tolerance test (OGTT) and an abbreviated version of the intravenous glucose tolerance test (IVGTT) were carried out in *n* = 57 Chilean nondiabetic women with measurements of plasma glucose, insulin, and C-peptide. Bisulfite-treated DNA from leukocytes was evaluated for methylation levels in six CpG sites of the proximal promoter of the PPARGC1A gene by pyrosequencing (positions -816, -783, -652, -617, -521 and -515). A strong correlation between the DNA methylation percentage of different CpG sites of the PPARGC1A promoter in leukocytes was found, suggesting an integrated epigenetic control of this region. We found a positive association between the methylation levels of the CpG site -783 with the insulin sensitivity Matsuda composite index (rho = 0.31; *p* = 0.02) derived from the OGTT. The CpG hypomethylation in the promoter position -783 of the PPARGC1A gene in leukocytes may represent a biomarker of reduced insulin sensitivity after the ingestion of glucose.

## 1. Introduction

Although physiologically essential, glucose is a nutrient that does not meet the necessary criteria for nutritional essentiality, except in rare genetic diseases affecting endogenous glucose production, such as glycogen storage disease type I [1]. As a nutrient, glucose appears in food as intrinsic glucose (e.g., in grapes), as free glucose (e.g., in honey), as part of disaccharides/oligosaccharides (e.g., sucrose, lactose and raffinose), attached to other dietary compounds (e.g., bound to the aglycone part of many dietary polyphenols) or being the monomer of polysaccharides of plant or animal origin (e.g., starch and glycogen). Starch, as the storage molecule of carbohydrates in plants, is considered one of the major contributors of energy intake in human diets [2,3], while current recommendations encourage reductions in added sugar consumption. Apart from a being a nutrient, glucose is fundamentally the center of a sophisticated homeostatic neuroendocrine system that controls the blood levels of this monosaccharide across the feed-fasting cycle. Type 2 Diabetes (T2D), the most common form of diabetes, is a multifactorial disease related to an inappropriate diet, which results from multi-organ dysfunction leading to hyperglycemia, the hallmark of all types of diabetes [4]. Moreover, circulating glucose levels can be viewed as an archetypal biomarker of T2D, given that glycemia is simultaneously used in disease diagnosis, used to monitor clinical management, and used in the prediction of disease complications (https://www.ncbi.nlm.nih.gov/books/NBK326791/) [5]. Given the dominant role of insulin in glucose homeostasis, it is stated that T2D results from the combination of reduced insulin sensitivity coupled with inappropriate insulin secretion [6]. Thus, both insulin secretion (occurring in pancreatic β-cells) and insulin sensitivity (occurring in insulin-sensitive tissues such as the liver, muscle and adipose), or combined indexes of insulin secretion/sensitivity, such as the Disposition Index, are important predictors for conversion to T2D [7,8]. Additionally, genetic markers associated with the differential management of dietary glucose could be also considered as biomarkers of glycemic responses after glucose challenges, and indirectly as biomarkers of future T2D. In spite of the importance of genetics in T2D development, Genome-wide association studies (GWAS) have shown that only a relative low percentage of heritability of T2D can be explained by common gene variants [9], although recent sophisticated statistical analyses have provided evidence of a higher predictive ability of genetic markers in disease prediction [10]. An alternative source of gene-related biomarkers relies on epigenetic events that occur in genes related to glucose and insulin metabolism. In this context, DNA methylation in different genes and tissues (including insulin-producing pancreatic β-cells, insulin-sensitive tissues and blood) have been proposed as the biomarkers of insulin secretion/sensitivity, biomarkers of glycemic responses after glucose challenges, or directly as biomarkers of T2D [11,12,13]. 

As a master regulator of many metabolic cell adaptations in a variety of tissues and physiologic contexts, the role of the peroxisome proliferator-activated receptor γ coactivator-1 alpha (PGC-1α, encoded by the PPARGC1A gene) has been extensively studied in the etiology of T2D [14]. PGC-1α belongs to a family of transcriptional coactivators that participate in the regulation of cellular energy metabolism [15]. PGC-1α has been involved in mitochondrial biogenesis, adaptation to exercise and fiber-type switching in skeletal muscle, cold-induced thermogenesis in brown adipocytes, adaptation to fasting, and the maintenance of glucose homeostasis [16]. The PPARGC1A gene has also been proposed to be epigenetically regulated by DNA methylation in response to dietary challenges such as high-fat overfeeding [17], episodes of physical exercise [18] and fasting [19]. In the liver, PGC-1α promotes fast-induced gluconeogenesis and fatty acid oxidation [20,21,22]. Several studies have shown that subjects with a family history of T2D display a low expression of PPARGC1A in insulin-sensitive tissues, such as muscle and adipose tissue [23,24], possibly affecting PGC-1α-responsive genes involved in oxidative phosphorylation [25]. In controversial studies, it was also proposed that PGC-1α overexpression in muscle fibers may enhance glucose uptake and improve insulin sensitivity, through the increased translocation of GLUT4 to the cell membrane [26,27]. It has been also described that PGC-1α-responsive genes involved in oxidative phosphorylation were coordinately downregulated in human diabetes muscle [25]. Regarding insulin secretion, PGC-1α seems to be also relevant in the modulation of pancreatic β-cell function [28]. Unlike its role in other tissues, PGC-1α seems to play a minor role in β-cell mitochondrial biogenesis, but it is important in potentiating the effect of non-esterified fatty acids on glucose-stimulated insulin secretion [28]. It was initially suggested that PGC-1α negatively regulated insulin secretion, given that its expression was elevated in islets of diabetic rodent models (ob/ob mice and Zucker diabetic fatty rats) [29]. Moreover, decreased insulin secretion was observed in islets from transgenic mice overexpressing PGC-1α [29,30]. However, a reduced PPARGC1A messenger RNA (mRNA) expression was also reported in islets from streptozotocin-nicotinamide diabetic male Wistar rats and NIDDM1I strain compared to controls [31]. Interestingly, it was found that the methylation level of the PPARGC1A promoter was two-fold higher in the pancreatic islets from T2D patients compared to non-diabetic donors [31], also showing an inverse correlation with insulin secretion capacity and a trend for lower PPARGC1A gene expression. In this latter research, the PGC-1α expression and DNA methylation levels of PPARGC1A were also dependent on genotypes of the missense polymorphism Gly482Ser (rs8192678), which has been repeatedly reported in association with T2D and insulin secretion/sensitivity indexes [32,33,34,35,36]. 

Different studies have associated methylation in the PPARGC1A gene with adiposity [37] and whole-body or tissue-specific insulin sensitivity/resistance [38,39,40]. Notably, the percentage of methylation of the CpG site at the position -783 of the PPARGC1A gene in muscle was significantly associated with measures of whole-body insulin sensitivity derived from the oral glucose tolerance test (OGTT) [38], being the only CpG site of PPARGC1A showing such significant association. Then, it would be interesting to test whether the percentage of methylation of this CpG site calculated from leukocytes (as a more accessible tissue compared to muscle) was also associated with whole-body insulin sensitivity indexes derived from OGTT. Other studies did not find association between CpG sites in the promoter region of PPARGC1A and insulin sensitivity/resistance indexes [41]. However, such studies did not include the CpG at position -783, since this site and other genomic positions in the PPARGC1A gene promoter are not included in the Illumina methylation arrays that are commonly used in epigenomic studies. 

To our knowledge, there are no studies that have determined the methylation levels of multiple CpG sites of the promoter region of the PPARGC1A gene in leukocytes using a gold standard method (pyrosequencing), and evaluate their relationship with insulin secretion/sensitivity indexes derived from oral and intravenous glucose challenges. Then, the aim of this study was to assess the association between the methylation levels of the PPARGC1A gene promoter in leukocytes by pyrosequencing (especially at the -783 position) with indexes of insulin secretion/sensitivity after glucose challenges in normoglycemic women.

## 2. Subjects and Methods

### 2.1. Design, Subjects and Anthropometric Measurements

A group of *n* = 57 non-obese, non-diabetic Chilean women from the GEDYMET cohort (Genetics, Dysglycemia and Metabolism) (age: 27.4 ± 6.9 years; body mass index (BMI): 23.8 ± 3.5 kg/m^2^) participated in this study [42]. In a first visit, biochemical and anthropometric measurements were carried out after overnight fasting, which included weight, height, blood glucose, insulin, total cholesterol, HDL cholesterol, triglycerides; and systolic and diastolic arterial pressure. Exclusion criteria were the diagnosis of diabetes, a family history of diabetes, dyslipidemia, anemia or pregnancy. Most participants (95%) were nulliparous, and none of them suffered from previous gestational diabetes. This research was conducted in the Centre of Clinical Research (School of Medicine, Pontificia Universidad Católica de Chile, Santiago, Chile) and approved by the Ethics committee of the School of Medicine, Pontificia Universidad Católica de Chile (Santiago, Chile) (code of the ethics committee: 11-132; consent approval date: 6 August 2013).

### 2.2. Insulin Secretion/Sensitivity Indexes Derived from the Oral Glucose Tolerance Test (OGTT)

After the screening visit described above, the participants visited our research center for a second time after approximately one week to carry out a standard 75 g OGTT after 8–12 h fasting, and drawing blood samples at times; −15, −5, 15, 30, 60, 90 and 120 min of the ingestion of the oral glucose load. Samples were used for the determination of plasma glucose, insulin and C-peptide levels. Venous blood samples were drawn in tubes containing sodium fluoride for glucose measurements and EDTA-containing tubes for biochemical and hormone determinations. Plasma and leukocytes were separated from whole blood by centrifugation at 3500 rpm, 5 °C, 15 min, and frozen immediately at −80 °C until assay. Plasma levels of insulin (µU/mL) and glucose (mg/dL) were measured by electro-chemiluminescence and colorimetric methods, respectively. C-peptide levels in plasma were measured by radioimmunoassay (ng/mL; DiaSorin Inc., Saluggia, Italy) at −15 and 30 min during OGTT. The Insulin Sensitivity Matsuda composite (Matsuda-ISICOMP) index was calculated from the OGTT [43]. Insulin secretion was determined by the insulinogenic index and the C-peptidogenic index (both at minute 30). The C-peptidogenic index is equivalent to the insulinogenic index using plasma C-peptide instead of insulin measurements [44]. OGTT-based Oral Disposition Indexes (ODIs), which are surrogates of insulin secretion adjusted by systemic insulin sensitivity, were calculated as previously described [42] and as the product of the Matsuda-ISICOMP index by the C-peptidogenic index.

### 2.3. Insulin Secretion/Sensitivity Indexes Derived from the Intravenous Glucose Tolerance Test (IVGTT)

In a third visit (approximately occurring one week apart from the second visit), the volunteers also carried out an abbreviated version of the minimal model intravenous glucose tolerance test (IVGTT). This procedure consisted of the intravenous administration of 0.3 g glucose per kg of body weight, as a 50% water solution, infused for 1 min [45]. Plasma glucose and insulin levels were measured at −15, −5, 2, 3, 4, 5, 6, 8 and 10 min to calculate the Acute Insulin Release (AIR) Index as the area under the curve of plasma insulin [46]. After AIR, additional glucose and insulin levels were measured in plasma obtained at 10, 15, 20, 30, 40 and 50 min, and used to estimate the Calculated Sensitivity Index (CSi) (see calculations at http://webmet.pd.cnr.it/csi/) [45]. The IVGTT-based Disposition Index (DI = AIR × CSi) (considered as a measure of insulin secretion adjusted by systemic insulin sensitivity) was also calculated. From fasting plasma measurements of glucose and insulin in the second and third visits, the homeostasis model assessment (HOMA)-S index, a surrogate measure of insulin sensitivity, was calculated as the averages of the values obtained in the second and third visits. The HOMA-S index is the inverse of the HOMA-IR index (HOMA-S = 1/HOMA-IR), being HOMA-IR = (1/(fasting insulin (µUI/mL) × fasting glucose (mg/dL))/405).

### 2.4. Hemogram and Differential Leukocyte Counts

In the OGTT visit, the presence of anemia was assessed through a complete hemogram, including hemoglobin and hematocrit, as well as the cell counts of erythrocytes, platelets, and white blood cells. Differential leukocyte counts (neutrophils, basophils, eosinophils, lymphocytes, and monocytes) were determined by automated hematology analyzers at the laboratory of Red-Salud UC-Christus (Santiago, Chile; https://agenda.saluduc.cl/Sinfex/). Counts of different white cell types will also be used to assess their association with DNA methylation levels in total leukocytes. All participants showed normal values in the hemogram. Apart from the white cell counts, the absence of overlooked infections was also screened by tympanic membrane temperature (all participants showed temperatures <37 °C).

### 2.5. Pyrosequencing and Array-Based Methylation Analysis of the Promoter Region of PPARGC1A Gene

Leukocyte DNA was extracted from whole blood using the Qiagen Mini-blood kits, fluorometrically quantified, and subsequently treated with sodium bisulfite (Epitect bisulfite kit, Qiagen, Hilden, Germany) to convert the unmethylated cytosine into uracil. The promoter region of the PPARGC1A gene was amplified by PCR using previously published primers [37]. One of the PCR primers included a 5′-biotin tag, allowing the isolation of the PCR product from the reaction mix. Then, the biotin-containing amplified DNA strand was submitted to a PyroMark Q96 MD pyrosequencer (Qiagen, Hilden, Germany) to determine the percentage of methylation in CpG sites in the canonical promoter region of PPARGC1A (-816, -783, -652, -617, -521 y -515, relative to the transcription start site—TSS) by comparing the signal intensity of non-bisulfite-sensitive CpG and bisulfite-sensitive CpG sites. 

The present study was also part of the Methyl Epigenome Network Association (MENA) project [47,48] (available at Gene Expression Omnibus (GEO); https://www.ncbi.nlm.nih.gov/geo/) (Accession number: GSE115278) in which a genome-wide DNA methylation profile of the GEDYMET volunteers was assessed using a methylation DNA array. In this assay, bisulfite-treated DNA was hybridized in an Illumina Infinium HumanMethylation450 BeadChip (Illumina, San Diego, CA, USA) following the standard manufacturer’s protocol and scanned using the Illumina iScanSQ platform. This analysis was conducted at the Unidad de Genotipado y Diagnóstico Genético from the Fundación de Investigación Clínico de Valencia. The intensity of the images was extracted with the GenomeStudio Methylation Software Module (v 1.9.0, Illumina). β-Values were computed using the formula β-Value = M/(U + M) where M and U are the raw “methylated” and “unmethylated” signals, respectively. β-Values were corrected for type I and type II bias using the peak-based correction and normalized in R (https://www.r-project.org/) using a categorical subset quantile normalization method. Five CpG sites of the canonical PPARGC1A promoter were retrieved from the Illumina 450K array: CpG at -921 (cg05158538), -841 (cg27182172), -816 (cg11270806), -383 (cg04893087), and -135 (cg12691631) (positions referred to the transcription start site of PPARGC1A). 

**Figure 1:**
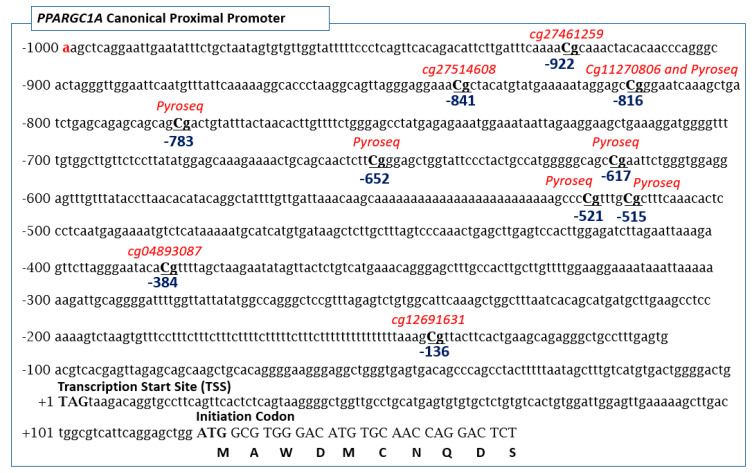
CpG sites in the PPARGC1A gene promoter assessed in this study.

In rodents and humans, the PPARGC1A gene shows canonical (proximal) and alternative (distal) promoters in such a way that the use of multiple promoters and alternative splicing produce a variable number of coactivator variants in different tissues. The most quoted isoform of PPARGC1A correspond to the long 798 amino acid showing a high degree of identity with the rodent sequence (Gene ID = 10891; NG_028250.2; NM_013261.5) [49]. Figure 1 shows the DNA sequence of the proximal promoter region of the PPARGC1A gene showing the CpG sites evaluated in this study. Note that the positions -961 to -746 relative to the translation start site reported by Ling et al. [31] are identical to the -841 to -617 positions relative to the transcription start site assessed in our study. Consequently, the -783-position reported herein is the same as the -902-position referred to by Ling et al. [31].

### 2.6. Determination of PPARGC1A Gly482Ser Genotypes

The common Gly482Ser polymorphism rs8192678 (NG_028250.2: g.663937G>A), changing GGT to AGT in the 482 amino acid position (Glycine to Serine) of PPARGC1A, was genotyped by polymerase chain reaction (PCR) followed by restriction fragment length polymorphism analysis (PCR–RFLP). The primers used were: 5′-AGGCAAGCAAGCAGGTCT-3′ (forward) and 5′-GTCATCAAACAGGCCATCC-3′ (reverse). The 224-bp PCR products were incubated with the MspI restriction enzyme at 37 °C for 4 h, resolved in 2.5% agarose gel electrophoresis and visualized with SYBR Safe stain (expected fragments for the heterozygous genotype: 224, 127 and 97 bp).

### 2.7. Statistical Methods

Data from the DNA methylation levels and metabolic variables were presented as the mean ± standard deviations. Association between the numerical variables were assessed through non-parametric Spearman correlation and linear regression. Receiver operating characteristic (ROC) curves were calculated to assess the capacity of the methylation percentages at CpG sites in the PPRGC1A gene to classify insulin-sensitive versus insulin-resistant subjects. Concordance between the measurements of methylation levels by pyrosequencing and Illumina 450K array was assessed by the Lin concordance coefficient. Allele and genotype frequencies of the Gly482Ser polymorphism were calculated, and agreement with Hardy–Weinberg proportions was assessed by a goodness-of-fit chi-squared test. Differences by genotypes (482Ser carriers versus non-carriers) were assessed by the Mann–Whitney test. Considering an allele frequency >0.3 for the 482Ser allele from the Spanish population [50], a sample size of *n* = 57 gives a statistical power of >90% (with 95% of confidence) to detect differences of 1 standard deviation in the Matsuda-ISICOMP index when comparing carriers versus non-carriers of the 482Ser allele of PPARGC1A. *p*-values < 0.05 were considered as statistically significant. All statistical analyses were carried out using STATA 14.0 program (StataCorp, College Station, TX, USA; www.stata.com).

## 3. Results

**Figure 2:**
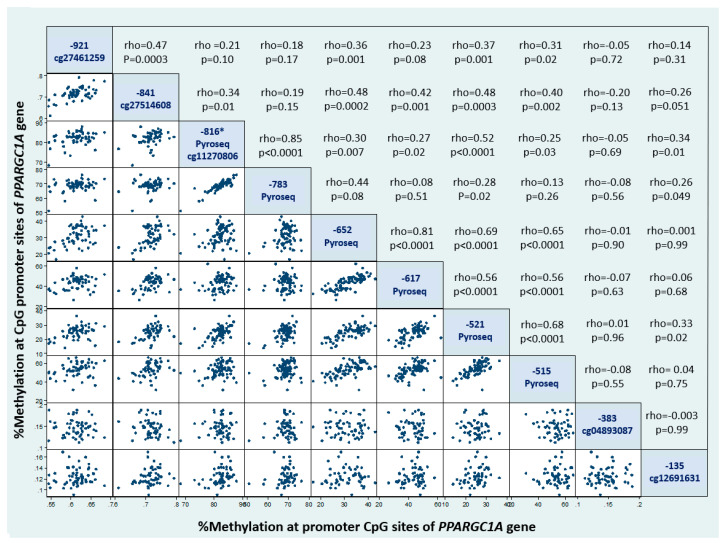
Scatter plots showing the methylation levels of CpG sites at the PPARGC1A promoter in leukocytes. * Pyrosequencing measurements were used for methylation levels at the -816 CpG position in this chart.

Table 1 shows the general characteristics of this study group in relation to the variables assessed in this research. Methylation levels for CpG sites measured by pyrosequencing in the canonical promoter of PPARGC1A were strongly positively correlated to each other, as well as associated with other methylation sites measured by the Illumina Methylation 450K array (Figure 2). The strongest association was found between the percentage of methylation at positions -816 and -783 (rho = 0.85; *p* < 0.0001: both assessed through pyrosequencing). We also found significant correlations between methylation levels in sites measured by pyrosequencing with those determined with the Illumina 450K array (Figure 2), except for the CpG site cg04893087 at the -384 position, which did not correlate with any of the other CpG sites. The percentage of DNA methylation at position -816 was determined both by pyrosequencing and using the Illumina 450K array (cg11270806). Both measurements were significantly correlated (rho = 0.44; *p* = 0.006), with a Lin concordance coefficient computed with z-scores of methylation percentages of rc = 0.46 (95%CI = 0.24–0.63; termed as a weak concordance). Moreover, the correlation between methylation in CpG cg11270806 (Illumina 450K array) and methylation at -783 (pyrosequencing) is non-significant, indicating that cg11270806 is a poor surrogate of the methylation level at -783 CpG site. However, the use of the cg11270806 probe from the 450K Illumina array as a surrogate of methylation at the -816 position was sill significantly associated with other CpG sites within the PPARGC1A promoter (*p* < 0.001), except for the aforementioned CpG at -783 (measured by pyrosequencing; rho = 0.19; *p* = 0.16), CpG at -384 (cg04893087; rho = −0.01: *p* = 0.94) and CpG at -136 (cg12691631; rho = 0.17: *p* = 0.20). 

**Figure 3:**
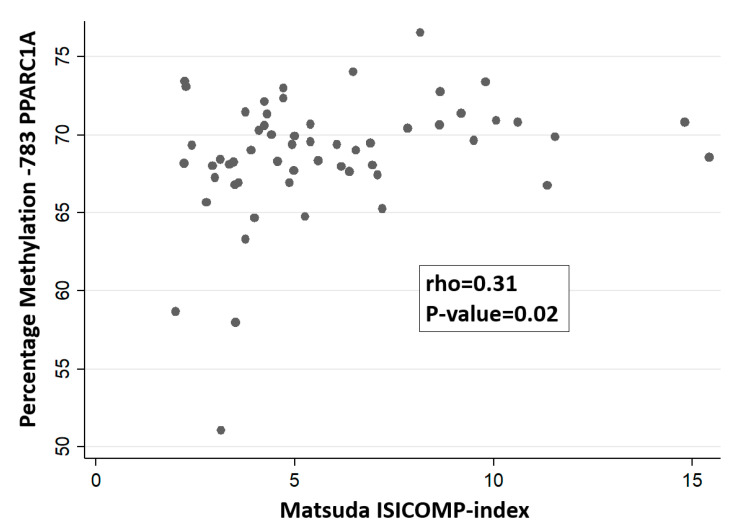
Association between the methylation levels in the -783 CpG position of the PPARGC1A gene in leukocytes with the Matsuda composite index.

**Figure 4:**
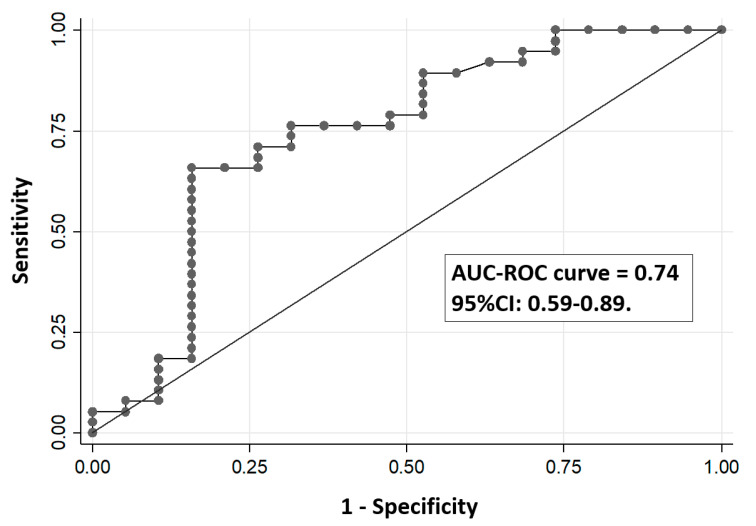
Receiver operating characteristic (ROC) curve to assess the capacity of the percentage of DNA methylation at the -783 CpG position of the PPARGC1A gene in leukocytes to classify insulin-sensitive versus insulin-resistant subjects. AUC: Area under the curve.

We analyzed all the possible correlations between the percentage of methylation of PPARGC1A promoter sites and insulin secretion/sensitivity indexes (including Disposition Indexes). We only found a nominal positive association between the methylation levels of the CpG site -783 (measured by pyrosequencing) with the Matsuda-ISICOMP index (rho = 0.31; *p* = 0.02) (Figure 3) with a near-significant association with ODI (rho = 0.27; *p* = 0.05). Such significant association agrees with our initial hypothesis indicating an involvement of methylation levels at the -783 CpG position in insulin sensitivity, although it would not have been significant after correction for multiple comparisons in an hypothesis-free study. Regression analysis using Matsuda-ISICOMP index as a dependent variable and methylation levels of the CpG site -783 as an independent variable showed a significant association between both variables (slope: 0.21; 95%CI: 0.02–0.40; *p* = 0.03) that remain nearly unchanged after the inclusion of BMI as an additional covariate in the regression model (slope: 0.21; 95% CI: 0.02–0.40; *p* = 0.03). No significant association was found between the CpG site -783 with CSi index from IVGTT (*p* = 0.76) or the HOMA-S index (*p* = 0.23). For the other CpG sites, none of them achieved nominal statistical significance when correlated with insulin sensitivity indexes (Matsuda-ISICOMP index, HOMA-S, the C-peptidogenic index, or the ODI), with *p*-values > 0.10 in all tested correlations. None of the % methylation of the CpG sites of the PPARGC1A gene promoter were significantly associated with insulin secretion indexes either from OGTT or IVGTT. Figure 4 shows the ROC curve assessing the capacity of the percentage of DNA methylation at the -783 CpG position of the PPARGC1A gene to classify the insulin-sensitive versus insulin-resistant subjects, defined as those below the 25th percentile of the Matsuda-ISICOMP distribution (area under the ROC curve = 0.74; 95%CI: 0.59–0.89).

We did not find significant associations between the methylation levels of the proximal promoter region of PPARGC1A with the total white cell count, or other white cell subtypes measured in blood samples (basophiles, eosinophils, neutrophils, lymphocytes and monocytes) (*p*-values > 0.1 in all associations). This lack of association indicates that there is a low chance of confounding effect due to the heterogeneity of leukocyte population counts in the assessment of the association between the methylation levels of the PPARGC1A gene with insulin secretion/sensitivity indexes.

The allele frequency of the 482Serine allele in our group was 0.30, this polymorphism being in agreement with the Hardy–Weinberg equilibrium (*p* = 0.92). Given that the frequency of the Ser/Ser genotype was low (only five subjects), the Ser482/Ser482 genotype was combined with the heterozygous Gly482/Ser482 and then compared with Gly482/Gly482 genotypes (carrier 482Ser versus non-carriers) for statistical analyses. The Mann–Whitney and *t*-tests did not detect significant associations between the genotypes of Gly482Ser and the methylation levels of the promoter region of PPARGC1A, either in the CpG sites assessed by pyrosequencing (all *p*-values above 0.32) or by the 450K Illumina array (all *p*-values above 0.21). Likewise, no significant associations were found between the Gly482Ser genotypes (rs8192678) with insulin secretion/sensitivity indexes derived from OGTT or IVGTT (all *p*-values above 0.2).

## 4. Discussion

We evaluated the association between the methylation levels of CpG sites in the canonical promoter region of the PPARGC1A gene in leukocytes with insulin secretion/sensitivity indexes in normoglycemic women derived from the OGTT or IVGTT. We detected a clear epigenetic methylation signature in the promoter region of the PPARGC1A gene characterized by strongly correlated methylation levels of different CpG sites, suggesting an integrated epigenetic control of this gene (Figure 2). There is suggestive evidence from the scientific literature indicating a relevant role of epigenetic marks of the PPARGC1A gene with insulin-related glycemic traits and adiposity, also considering that DNA methylation in leukocytes may mirror methylation patterns in insulin-sensitive tissues [31,37,38,39,40]. However, most studies assessing epigenetic biomarkers related to insulin secretion/sensitivity indexes have been based on methylation levels derived from the Illumina 450K array [31,38,40,41]. Despite its wide genome coverage, the Illumina Methylation 450K array under-represents critical CpG sites at the proximal promoter region of PPARGC1A. In agreement with a previous study carried out in muscle [38], we found herein that the hypomethylation of the promoter site -783 CpG of the PPARGC1A gene in leukocytes measured by pyrosequencing (not included in the Illumina Methylation 450K array) might represent a biomarker of reduced insulin sensitivity (or enhanced insulin resistance) (Figure 3 and Figure 4). In an exploratory analysis, we also found that genotypes of the common polymorphism Gly482Ser were not associated with the methylation levels of PPARGC1A promoter gene or with insulin secretion or sensitivity indexes. 

Multiple evidence indicates that PPARGC1A is a methylation-sensitive gene in diverse tissues and organs involved in glucose homeostasis such as pancreatic insulin-producing β-cells, pancreatic islets, and insulin-sensitive tissues [19,31,38,39,40]. Our results regarding the nominally-significant association between the percentage of methylation at the CpG site -783 of PPARGC1A with the Matsuda-ISICOMP index are concordant with a previous research in which the methylation levels at this CpG site in muscle was significantly associated with the Matsuda-ISICOMP insulin sensitivity index [38], being the only CpG site showing a significant association. Additionally, Gillberg et al. [38] also found lower (although not significant) methylation levels in diabetic patients compared to normoglycemic subjects. It was reported that a sequence near the -783 position lies in a predicted hepatic nuclear factor 1 binding site (HNF-1) [31] and a PBX-1/HOXB9 response element, which might be potentially implicated in lipid and glycemic traits related to adiposity and T2D [37]. Importantly, it was found that the methylation level at the -783 position alters the transcription factor binding capacity using electrophoretic mobility shift assays in nuclear extracts from liposarcoma cells [37]. The latter result indicates that the position -783 in the promoter region of the PPARGC1A gene plays a relevant role in regulating its transcriptional activity. In this sense, the proximal promoter region of PPARGC1A which includes the -783 CpG site contains a number of sequence motifs with putative transcription factor binding sites that may affect gene expression [31,37], as predicted by the online programs TRANSFAC (14 transcription factors binding sites with score >0.95, including BEN, NFAT-related factors, FOXC) (http://www.genexplain.com) and the program MatInspector (10 predicted transcription factor binding sites with a matrix similarity >0.99) (http://www.genomatix.de). In this sense, a non-significant trend for an inverse association between the average DNA methylation at the PPARGC1A gene promoter with an expression was reported in pancreatic islets [31]. In contrast, a direct significant association between methylation levels in the promoter region of PPARGC1A and gene expression was found in the muscle [38]. Interestingly, it was also reported that the exercise-induced hypomethylation of PPARGC1A promoter is accompanied by an increased gene expression in skeletal muscle [18]. Apart from the aforementioned reports, the association between the level of methylation in the CpG sites of PPARGC1A and its gene expression can be also assessed using the MEXPRESS online utility (https://mexpress.be/) based on The Cancer Genome Atlas [51]. A limitation of this online tool is that it only contains data on CpG sites measured with the Illumina arrays, not having data of the -783 CpG site. Using MEXPRESS, we specifically examined the correlation between methylation at the cg11270806 probe (located at the -816-position relative to the PPARGC1A transcription start site, being the nearest probe to the -783 CpG site) with PPARGC1A expression in different cancer types. It was found that cg11270806 was inversely associated with gene expression in 14 cancer types, directly associated in two cancer types, and showed no significant correlation in another 16 cancer types. Although inconclusive, the data from MEXPRESS might suggest that the hypermethylation of the CpG sites of the PPARGC1A gene promoter is often associated with a lower gene expression. 

We previously reported that the methylation levels of CpG sites in the PPARGC1A gene measured through Illumina 450K array did not reach significant association in relation to insulin sensitivity measured as HOMA-IR (from fasting blood samples) or CSi (from IVGTT) in the participants of the present study [47,48]. Using peripheral blood mononuclear cells as a source of DNA, another epigenome-wide study compared insulin-resistant versus insulin-sensitive non-diabetic women with obesity, classified according to hyperinsulinemic euglycemic clamps, without finding differences by PPARGC1A methylation status [41]. However, such studies (available at GEO; Accession number: GSE76285) did not include insulin sensitivity measures derived from OGTT and were limited to CpG methylation sites contained in the Illumina 450K array (not including the -783 CpG position). In the present report, we evaluated CpG sites in the PPARGC1A promoter evaluated by pyrosequencing, considered as the gold standard for DNA methylation analysis. The restricted association found in our study between the -783 methylation site of PPARGC1A with Matsuda-ISICOMP index (derived from OGTT), and not with CSi (derived from IVGTT) or HOMA-S (based on fasting blood samples) suggest that this epigenetic biomarker might be also related with factors involved in postprandial glycemia (e.g., incretin action, glucose digestion/absorption or intestinal gluconeogenesis) and possibly also connected to muscle insulin sensitivity. In spite of the recognized modest sample size of our study, we must emphasize that our research is unique from the point of view that the are no studies with a simultaneous assessment of insulin secretion and sensitivity indexes based on OGTT and IVGTT in relation to PPARGC1A methylation levels (measured by the gold standard pyrosequencing). Interestingly, none of the CpG sites assessed in our study associated with insulin secretion indexes (either based on C-peptide from OGTT or AIR from IVGTT) indicated that the methylation of the PPARGC1A gene in leukocytes is not an adequate biomarker of insulin secretion. 

Our research has several strengths and limitations. This study was carried out in a relatively homogeneous group of participants, in terms of age, BMI and glycemic status (young adult normoglycemic women without obesity). Although the homogeneity of subjects limits the number of possible confounding factors, it also restricts the range of variability of the study participants in critical variables related to energy and glucose homeostasis. In this context, the hypothetical recruitment of participants with overweight or obesity would have expanded the variability in terms of insulin sensitivity indexes, given the well-known relation between adiposity and insulin resistance. In our study, the association between methylation levels in the -783 CpG site with the Matsuda-ISICOMP index was independent of BMI. However, additional studies should be carried out to assess the performance of this epigenetic biomarker in groups of different degrees of obesity and diabetes status. It is also important to mention that the present study was designed to develop biomarkers of insulin sensitivity in young healthy women, with the final aim of using such biomarkers in population-based epidemiologic studies that we are conducting in Chile [52]. An advantage of this study was the use of insulin secretion/sensitivity based on glucose challenges, instead of surrogate measurements based on plasma fasting samples such as HOMA-IR. Likewise, the use of gold-standard techniques for assessing DNA methylation is a strength of this study. In contrast, reduced sample size is a limitation. Another limitation is that we did not consider possible plasma insulin fluctuations due to differences in phases of the menstrual cycle. Finally, heterogeneity in the proportion of blood cell types may represent a confounding factor when assessing the association between DNA methylation levels as blood biomarkers in relation to traits [53]. Similar to previous reports [37], we did not find associations between the methylation levels of the proximal promoter region of PPARGC1A with a total white cell count, or with counts of white cell subtypes (basophiles, eosinophils, neutrophils, lymphocytes and monocytes). These results suggest a reduced confounding effect of a white cell-type population heterogeneity in the conclusions of our study [37].

We additionally explored the possible role of the common Gly482Ser polymorphism of the PPARGC1A gene in insulin secretion/sensitivity, given that this gene variant has been associated with T2D and glycemic traits, with diverse or even opposite effects in different studies [32,34,35,54,55,56,57,58]. The 482Ser variant gene produces a structural change in the PGC-1α protein that may have potential effects on its expression, compromising PGC-1α functions in different tissues. As shown by several studies, carriers of the 482Ser allele (Gly/Ser or Ser/Ser genotype) showed lower PPARGC1A mRNA expression compared with carriers of the Gly/Gly genotype [59]. Given that there is evidence indicating that a reduced expression of PGC-1α in endocrine pancreas may enhance glucose-stimulated insulin secretion, while a decreased expression of this gene may promote insulin resistance, it is possible to envisage an effect on this polymorphism in whole body insulin sensitivity. In the Diabetes Prevention Program trial, the 482Ser allele was associated with an accumulation of subcutaneous adiposity and worsening insulin resistance at 1-year lifestyle modifications including metformin and exercise [60]. Other studies, such as the STOP-NIDDM trial, found that this polymorphism was associated with a 1.6-fold higher risk of conversion from impaired glucose tolerance to diabetes [36]. As PPARGC1A mRNA expression is potentially affected by the Gly482Ser genotype and is also affected by CpG levels of methylation in the promoter region of the gene, we wonder whether Gly482Ser is associated with the methylation levels of the CpG site in the promoter region of the gene. With the assumed drawback in our limited sample size (only five subjects were homozygous for the Ser483 allele), we did not find evidence of such association. 

In conclusion, we found a tight correlation between the methylation percentage of different CpG sites of the PPARGC1A promoter gene in leukocytes, suggesting an integrated epigenetic control of this region. We also found that the specific hypomethylation of the promoter site -783 CpG of the PPARGC1A gene, measured though pyrosequencing, may represent a biomarker of reduced insulin sensitivity after the ingestion of glucose and a possible predictor of future T2D risk.

## Figures and Tables

**Table 1:** Study variables assessed in the non-diabetic Chilean women (*n* = 57) of this research.

	Mean ± Standard Deviation
Age (years)	27.4 ± 6.9
Weight (kg)	60.1 ± 9.3
Height (m)	1.59 ± 0.07
BMI (kg/m^2^)	23.8 ± 3.5
HOMA-S index	77.9 ± 44.0
OGTT-based Matsuda-ISICOMP index	5.7 ± 3.0
OGTT-based insulinogenic index	0.43 ± 0.22
OGTT-based C-peptidogenic index	0.31 ± 1.34
OGTT-based Disposition Index (ODI using C-peptide)	0.20 ± 0.12
OGTT-based Disposition Index (ODI using insulin)	3.06 ± 1.14
IVGTT-based CSi Index	6.46 ± 4.01
IVGTT-based AIR Index	59.7 ± 33.62
IVGTT-based Disposition Index (DI)	337.3 ± 202.4
% methylation (position -922) (Illumina 450K array)	61.5 ± 3
% methylation (position -841) (Illumina 450K array)	71.7 ± 3
% methylation (position -816) (Pyrosequencing)	82 ± 3.1
% methylation (position -783) (Pyrosequencing)	68.7 ± 3.6
% methylation (position -652) (Pyrosequencing)	31.5 ± 5.6
% methylation (position -617) (Pyrosequencing)	43.3 ± 6.6
% methylation (position -521) (Pyrosequencing)	24.8 ± 4.3
% methylation (position -515) (Pyrosequencing)	53.1 ± 7.0
% methylation (position -383) (Illumina 450K array)	14.7 ± 0.02
% methylation (position -136) (Illumina 450K array)	12.4 ± 0.02

AIR: Acute insulin release, BMI: Body mass index, CSi: Calculated sensitivity index, DI: Disposition Index, HOMA-S: Homeostasis model assessment-insulin sensitivity, IVGTT: Intravenous glucose tolerance test, Matsuda-ISICOMP index: Matsuda composite index, ODI: Oral disposition index, OGTT: Oral glucose tolerance test.
